# Equity in the distribution of CT and MRI in China: a panel analysis

**DOI:** 10.1186/1475-9276-12-39

**Published:** 2013-06-06

**Authors:** Da He, Hao Yu, Yingyao Chen

**Affiliations:** 1 Department of Hospital Management, School of Public Health, National Key Lab of Health Technology Assessment, Fudan University, Shanghai, 200032, P. R. China; 2 RAND Corporation, Pittsburgh, USA

**Keywords:** High technology medical equipment, Equity, Distribution, Financing, Lorenz curve, Gini coefficients

## Abstract

**Introduction:**

China is facing a daunting challenge to health equity in the context of rapid economic development. This study adds to the literature by examining equity in the distribution of high-technology medical equipment, such as CT and MRI, in China.

**Methods:**

A panel analysis was conducted with information about four study sites in 2006 and 2009. The four provincial-level study sites included Shanghai, Zhejiang, Shaanxi, and Hunan, representing different geographical, economic, and medical technology levels in China. A random sample of 71 hospitals was selected from the four sites. Data were collected through questionnaire surveys. Equity status was assessed in terms of CT and MRI numbers, characteristics of machine, and financing sources. The assessment was conducted at multiple levels, including international, provincial, city, and hospital level. In addition to comparison among the study sites, the sample was compared with OECD countries in CT and MRI distributions.

**Results:**

China had lower numbers of CTs and MRIs per million population in 2009 than most of the selected OECD countries while the increases in its CT and MRI numbers from 2006 to 2009 were higher than most of the OECD countries. The equity status of CT distribution remained at low inequality level in both 2006 and 2009 while the equity status of MRI distribution improved from high inequality in 2006 to moderate inequality in 2009. Despite the equity improvement, the distributions of CTs and MRIs were significantly positively correlated with economic development level across all cities in the four study sites in either 2006 or 2009. Our analysis also revealed that Shanghai, the study site with the highest level of economic development, had more advanced CT and MRI machine, more imported CTs and MRIs, and higher government subsidies on these two types of equipment.

**Conclusions:**

The number of CTs and MRIs increased considerably in China from 2006 to 2009. The equity status of CTs was better than that of MRIs although the equity status in MRI distribution got improved from 2006 to 2009. Still considerable inequality exists in terms of characteristics and financing of CTs and MRIs.

## Introduction

China is facing a daunting challenge to health equity in the context of rapid economic development. After China started its market-oriented economic reform in 1978, the centrally planned health care system, which emphasized universal basic health care for everyone, was transformed into a heavily market-based system with government decentralization [1,2]. As a result, health equity (as defined by prior studies [1,3]) has become a growing concern among policymakers and researchers. While the published research has reported on inequities in health status [1,4], health care [1,4,5], health insurance [6], and health care workforce [7], only a small number of studies have focused on equity in distribution of high-technology medical equipment, such as Computed Tomography (CT) and Magnetic Resonance Imaging (MRI). For example, two studies found that Gini coefficients of both CTs and MRIs were smaller than 0.35 which indicated a relatively equitable allocation of these two types of equipment across China. One limitation of these two studies is that they examined the diffusion of CTs and MRIs before 2004 [8,9] and do not reflect the new developments of recent years. There are six other studies of the diffusion state of CTs and/or MRIs, each of which was conducted in one specific province or city at one time point before 2007 [10-16]. Overall, there is a lack of information about whether the distribution of CTs and MRIs has become more equitable across China in recent years. Particularly absent are studies that examine the CT and MRI distributions by types of machine and sources of financing. It is important to analyze the distributions by types of machine because the advanced models of machine help improve the accuracy and speed of clinical diagnoses by providing higher quality medical images. Given the huge gap in socio-economic development across provinces in China, one question naturally arises of whether governments in those provinces with higher socio-economic development level have subsidized hospitals for purchasing CT and MRI, especially the machine with higher price. This study aims to fill the gap by comparing CT and MRI distribution in 2006 and 2009 in multiple provinces that represent different geographic regions and economic levels in China.

Examining equity in distribution of CTs and MRIs in China is particularly important for several reasons. First, after their introduction into the field of medical imaging in the 1970s [17], both CTs and MRIs have become widely used all over the world. In particular, unnecessary allocation and overuse of these two types of high-technology medical equipment have been known as part of the medical arms race in industrialized countries [18,19]. Given the rapid economic growth in China with the world’s second largest economy, some experts were concerned that a similar phenomenon of medical arms race may occur in China although they have not conducted empirical analyses to quantify their concerns [2].

Second, availability of health care resources, including CT, MRI, and other types of high-technology medical equipment, is important in defining health care systems’ performance. In recent years, international organizations, such as the World Health Organization (WHO) and the Organization for Economic Cooperation and Development (OECD) have emphasized the necessity of measuring equity in distribution of health care resources in addition to the traditional measurements of health care quality [20-22]. International studies have examined equity status of health care resource (including CT and MRI) distribution. One study found that the UK had relatively low MRI number per capita than other industrialized countries, and that the West Midlands region of the UK had less than the national average level of MRI number per capita [23]. A study in the USA found different diffusion patterns for CT and MRI with Medicare’s reimbursement policies and states’ certificate of needs (CON) regulation restricting MRI diffusion [24]. After the rise of managed care in the USA, researchers noted that MRI adoption responded positively financial and other incentives associated with managed care [25]. Similarly, one analysis of all thirty OECD countries concluded that diffusions CT and MRI were positively correlated with total health expenditure per capita and economic incentives to hospitals [26]. Researchers from developing countries have also paid attention to CT and MRI distribution. One study in Brazil found that MRI and CT diffusions were positively associated with average state income, and the inequalities had become larger over time [27]. However, there are few studies examining MRI and CT distribution across regions in China.

Third, there is a huge gap in socio-economic development among eastern, middle and western regions of China. Because the eastern region has more health care resources, an arms race among hospitals may exacerbate the equity problem in China’s allocation of health care resources across regions.

Fourth, Chinese hospitals, of which most are nominally owned by the government, started to receive less government investment after the economic reform in 1978. As a result, they have increasingly relied on prescribing expensive pharmaceuticals and diagnostic tests through high-technology medical equipment, such as CTs and MRIs, to balance their budgets [2,28]. This financial reason is critically important in understanding the wide adoption of CTs and MRIs by Chinese hospitals. One study reported that in China the number of CTs increased 55.4 percent and the number of MRIs increased 90.2 percent from 2002 to 2005 [2]. While that study used national data to examine the increases in CT and MRI numbers, it did not use the ratios of CTs and MRIs per million population to assess equity status of CT and MRI distributions. It also did not examine specific characteristics of CTs and MRIs, such as types and versions of the machines. While the newer versions of the equipment provide higher quality images quickly at higher prices [29,30], they also raise issues of equity regarding which hospitals purchased them and whether the purchase was subsidized by the government. The empirical literature provides little evidence on these issues.

Last but not least, in response to the heavy investment in high-technology medical equipment by hospitals, China’s central government implemented a policy in 2005, called Chinese certificate of needs (CON) [31], which aims to improve “appropriate allocation and efficient use of large medical equipment” through regional health planning and quota control. Under the policy, the Ministry of Health (MoH) has control over the total volume of major medical equipment, including CT and MRI; the provincial departments of health developed their regional plans and their detail regulations under the quota set by the MoH; and hospitals have to get a CON and apply for the quota scheme from provincial health departments before purchasing such equipment. It remains an unsettled question whether implementation of the policy results in reasonable growth and more equitable distributions of CT and MRI.

This study aims to examine the issues using panel data from multiple provinces in 2006 and 2009, which provide information not only about the numbers of CTs and MRIs but also detailed characteristics about these types of equipment such as machine model, price, and government subsidies for purchasing the machine. To inform China’s policymaking on allocation of CTs and MRIs, the study also compares the growth of the high-technology equipment between the study provinces and a group of selected industrialized countries. To our knowledge, this study is the first evaluation of inequality in CT and MRI distributions in terms of detailed characteristics of these two types of equipment. Our analysis is also the first study of how CT and MRI distributions are related to economic development and government subsidies in China.

## Methods

### Sample and data collection

We selected our study samples using three steps. First, we selected our study sites to reflect the huge variations in socio-economic development across regions in China. The selection of Shanghai, Zhejiang, Hunan, and Shaanxi as the study sites was based on the Chinese government’s official classification of regions. Shanghai was selected among China’s four mega-municipalities (Beijing, Shanghai, Tianjin, and Chongqing) to represent the most advanced socio-economic development and the highest level of medical technology in China while Zhejiang, Hunan, and Shaanxi were selected to indicate the east, middle, and west part of China. The rank of per capita gross domestic product (GDP) of Shanghai, Zhejiang, Hunan, and Shaanxi provinces was first, fifth, twentieth, and sixteenth respectively in 2009 among the 31 provincial administrative regions of China.

Second, within each of the three study provinces, two cities were selected from each province. While the capital city was selected to represent the highest level of medical technology in the province, a prefecture-level city with medium size and medium economic development level was selected to represent the “average” medical technology level in the province. Together with Shanghai, seven cities were selected. Within each city, we applied the systematic sampling method to select 25% of the secondary and tertiary hospitals respectively. The hospital sample consists of 71 hospitals. The sample does not include any primary hospitals because they are rarely equipped with CTs or MRIs.

Third, for each sampled hospital, all the CTs and MRIs were selected for data collection as described below.

The study period includes two years: 2006 and 2009. The year of 2006 was selected to reflect the situation when the above government policy on large medical equipment was implemented in 2005. The year 2009 was selected because it is the most recent year for which data were available at the time of conducting this study. The year 2009 also reflects the first year of the new round of health care reforms in China.

Data were collected from two sources. First, from the provincial-level Department of Health in each of the four study sites, a questionnaire survey was conducted to collect geographic, demographic and economic data, and numbers of CTs and MRIs. The data were collected both for the entire province and for each city within the province.

Second, from the sampled hospitals, detailed data about CTs and MRIs were collected using a questionnaire designed by the research team. The data included information about different types of machines, manufacturers, prices, and government subsidies for purchasing these machines.

Finally, for an internationally comparative analysis, the numbers of CTs and MRIs in some Organization for Economic Co-operation and Development (OECD) countries were obtained from the OECD official website [32]. Because not all the OECD countries had data available, only the 14 countries with available data were selected.

### Statistical analysis

#### Numbers and growth rates of CTs and MRIs

We analyzed the numbers of CTs and MRIs per million people in 2009 in the study sites and the selected OECD countries, and compared the growth rates of these two types of equipment from 2006 to 2009 between the study sites and the selected OECD countries.

#### Equity in the numbers of CTs and MRIs

We used Lorenz curves and Gini Coefficients to indicate equity status of CT and MRI distributions across the study provinces and across cities within each province. First, we used the cumulative proportion of a population and the cumulative distribution of CTs and MRIs to draw a Lorenz curve. This method was developed in the field of economics [33-36] and has gained increasing acceptance in public health and epidemiology [37,38]. In our study, a Lorenz curve represents the cumulative proportion of a population’s number of CTs and MRIs as a function of the cumulative proportion of the population, where the population is ranked from the lowest to the highest number. The situation of perfect equality—the case in which every person has the same number of CTs and MRIs in any region—is shown by the diagonal line. In general, a Lorenz curve lies below the diagonal, and the closer the curve gets to the diagonal line, the better the equity status.

We then used the Gini Coefficient to measure inequalities in the distribution of CTs and MRIs per million population. The Gini Coefficient is a summary measure that captures the deviation shown in the Lorenz Curve between observed and expected distributions. Its values can range between 0 and 1 with higher values indicating greater inequality [39,40]. Based on prior studies [41,42], a Gini coefficient smaller than 0.2 means low inequality level; between 0.2 and 0.3 moderate inequality; between 0.3 and 0.4, high inequality; higher than 0.4, extreme inequality [41,42]. While the Lorenz curve offers a visual description of the equity status, the Gini coefficients can show exactly how the equity status changes across regions over the years. Using the Gini coefficients, we will be able to not only separately analyze the equity status of CTs or MRIs but also examine the question of whether CTs are more equitably distributed than MRIs.

#### Association between economic development and numbers of CTs and MRIs

We assessed the relationship between diffusion of high-technology medical equipment and economic development by examining how the numbers of CTs and MRIs are correlated with a city’s per capita GDP across cities in the study sites.

#### Equity in the distributions of CTs and MRIs by characteristics of machine and sources of financing

Among the sampled hospitals, we assessed the distributions of CTs and MRIs in terms of machine types, manufacturers, prices, and government subsidies. For types of CT machines, we set two categories with one category of below 16-row^a^ and the other category of 16-row and higher CT, because CTs with more than 16 rows of scan per minute are with improved sensitivity [43]. For types of MRI machines, we also set two categories with one category of lower than 1.5T^b^ MRI and the other category of 1.5 T or higher level MRI, because MRI is most commonly performed at 1.5 T [29].

For manufacturers, we examined two groups: imported versus domestically produced CTs and MRIs. Domestic manufacturers in China did not produce CTs and MRIs until the late 1990s. Although domestically-produced machines are relatively less expensive than imported machines, hospitals generally prefer the imported machines because of their higher quality [44,45].

For prices, we examined whether the average prices of CTs and MRIs vary significantly across the study sites.

For government subsidies, we examined the numbers of CTs and MRIs with government subsidies and the proportions of government subsidies to total costs of CTs and MRIs among the study sites.

We conducted *χ*^2^ test to examine categorical variables, such as the distribution of imported CTs versus domestically produced CTs, while 95% confidence intervals were calculated for continuous variables, such as the prices of CTs. All of the above statistical analyses were produced by SPSS.

## Results

### Summary of the study sites

As shown in Table 1, there are substantial geographic, demographic, and economic differences among the study sites. Shanghai has the highest population density and lowest rural population percentage, and it was ranked first in GDP per capita among the four study sites in 2009. In comparison, Shaanxi and Hunan can be considered as large rural provinces, each of which has an area of more than 20, 000 km^2^, with half of its residents living in rural area. Overall, the sample is representative of the economic and urbanization trends in China.

**Table 1:** Description of the Study Sites in 2009

**Study site**	**Shanghai**	**Zhejiang**	**Shaanxi**	**Hunan**
Location	Eastern China	Eastern China	Northwest China	Central China
Number of cities within site	/	14	13	17
Area (10,000 km^2^)	0.63	10.18	20.58	21.18
Population (million)	19	47	38	69
Population density (person/km^2^)	3,049	463	183	326
Percentage of rural population of total population (%)	11.4	42.1	56.5	56.8
GDP per capita (CNY)	78,326	48,196	21,659	19,479
Rank of GDP per capita	1	5	16	20

### Numbers of CTs and MRIs in the study sites and the selected OECD countries

The upper part of Table 2 shows CT and MRI distributions among the study sites in 2009. The difference in the number of CTs per million population in 2009 across the study sites was relatively small, ranging from 6.03 in Hunan to 8.29 in Zhejiang. In comparison, there were large differences in the number of MRIs in 2009, with the highest number being 3.23 MRIs per million people in Shanghai which is more than double the number in Hunan.

**Table 2:** Number of CTs and MRIs per million population in the study sites and the selected OECD countries

**Study sites in China**	**CT**		**MRI**	
	**2009**	**Change from 2006 to 2009 (%)**	**2009**	**Change from 2006 to 2009 (%)**
Shanghai	7.6	62.7	3.2	72.8
Zhejiang	8.3	31.0	2.6	36.5
Shaanxi	8.5	63.8	2.2	63.7
Hunan	6.0	42.2	1.3	67.1
Entire sample	7.4	45.3	2.1	55.7
**OECD Countries**				
Australia	38.8	−30.7	5.9	22.9
Austria	29.9	1.0^*^	18.0	11.1
Canada	13.9	15.8	8.0	29.0
Denmark	23.7	50.0	15.4	51.0^†^
Finland	20.5	38.5	16.2	10.2^*^
Greece	33.9	28.4	21.8	33.7
Ireland	15.8	23.4	12.3	21.4
Israel	7.7	42.6	1.7	21.4
Iceland	34.5	42.6	21.9	11.2
Japan	97.3	5.1^‡^	43.1	7.5^*^
New Zealand	14.6	18.7^‡^	9.7	10.2^‡^
R.O.Korea	37.1	10.1	19.0	39.7
UK	7.4	−1.3^*^	5.6	3.7^*^
US	34.3	6.5^§^	25.9	−2.6^§^
Entire sample	41.8	1.04	24.1	−0.14

Note: *: change from 2005 to 2008, †: change from 2004 to 2009, ‡: change from 2007 to 2009, §: change from 2004 to 2007.

The numbers of CTs and MRIs per million population increased substantially for the entire sample with an average increase of 45.3% and 55.7% for CTs and MRIs respectively. The increases varied substantially across the study sites. Shaanxi experienced the largest increase in CT (64%) between 2006 and 2009, more than double the increase in Zhejiang (31%). Zhejiang also had the smallest growth in the number of MRIs between 2006 and 2009 (37%), considerably lower than other regions (73%, 64%, and 66% of increase for Shanghai, Shaanxi, and Hunan respectively). For each of the study sites, the increase of CTs from 2006 to 2009 was relatively slower than that of MRIs.

The lower part of Table 2 shows CT and MRI distributions in the selected OECD countries. Among the selected countries, Japan had the highest number of CTs per million population in 2009 (97.3) while the U.K. had the lowest level (7.4). In comparison, the study sample in China had 7.36 CTs per million people in 2009, even lower than that of the U.K.

Most of the selected OECD countries had a growing number of CTs between 2006 and 2009 except Australia and the U.K., both of which had negative growth of CTs. In comparison, all the study sites in China had a positive growth of CTs during 2006–2009, and the growth rate was higher than most of the selected OECD countries.

Table 2 also shows that the number of MRIs in the Chinese sample was 2.06 per million population in 2009, which was lower than each of the selected OECD countries except Israel (1.7 MRIs per million people). As the highest level of MRI allocation, Japan had 43.1 MRIs per million people, 20.9 times higher than the study sample in China. Among the selected OECD countries, there was a substantial variation in the increase of MRIs between 2006 and 2009 with Denmark having the largest increase (51.0%) and the U.S. having the largest decrease (−2.6%). In comparison, the study sample in China had an increase of 55.7% in the number of MRIs per million people between 2006 and 2009, higher than each of the selected OECD countries.

### Equity assessment across all the cities in the four study sites

**Figure 1:**
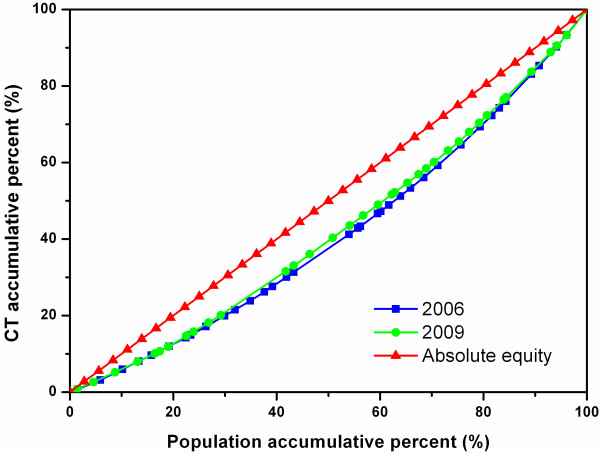
Lorenz Curve of CTs in all sample cities.

Figure 1 is the Lorenz Curves showing the relationship between population and the distribution of CTs across all cities in the four study sites. While most of the two curves overlapped, the 2009 curve was slightly closer to the diagonal line than the 2006 curve, indicating a slight improvement in the equitable diffusion of CTs.

**Figure 2:**
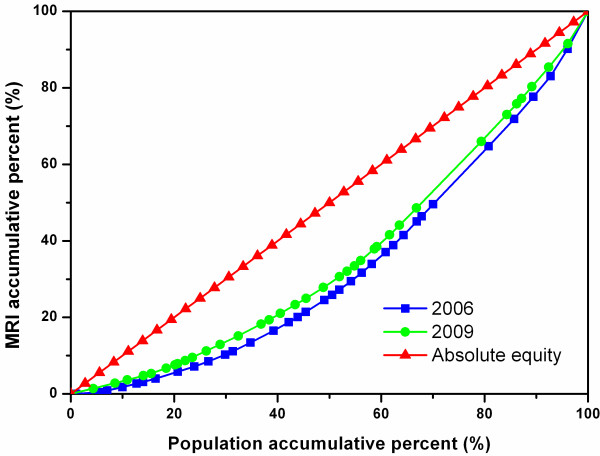
**Lorenz Curve of MRIs in all sample cities*.***

Figure 2 shows the Lorenz Curves of MRIs in all the cities in the four study sites. The 2009 curve was closer to the diagonal line than the 2006 curve, and the two curves were clearly separate from each other, reflecting a noticeable improvement in the equitable diffusion of MRIs between the two years.

### Equity assessment across cities within each of the study sites

**Figure 3:**
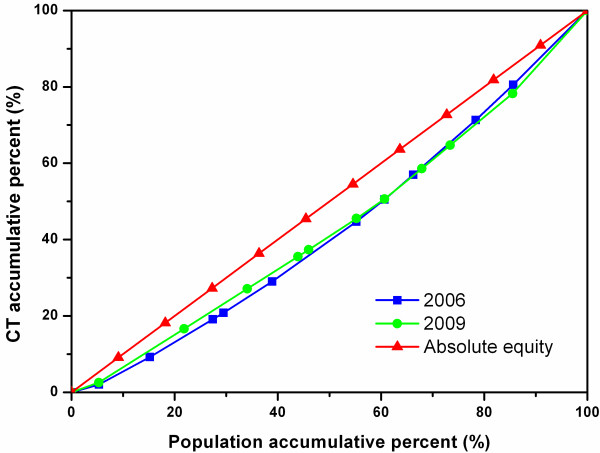
Lorenz Curve of CTs in all cities of Zhejiang.

**Figure 4:**
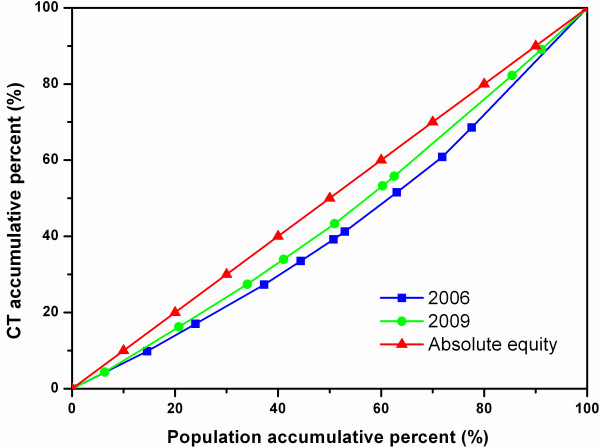
Lorenz Curve of CTs in all cities of Shaanxi.

**Figure 5:**
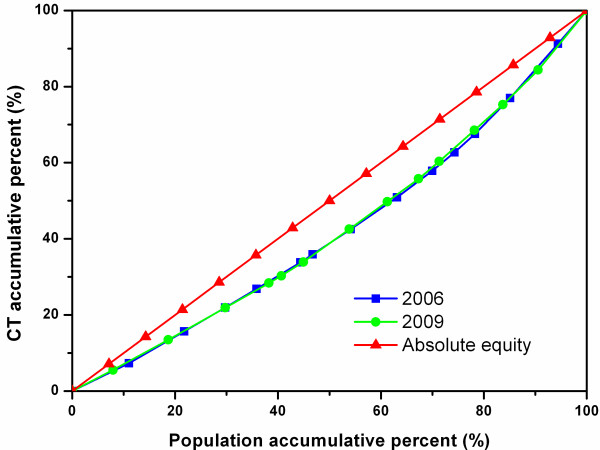
Lorenz Curve of CTs in all cities of Hunan.

Figures 3, 4, and 5 show the diffusion of CTs between 2006 and 2009 in Zhejiang, Shaanxi, and Hunan, respectively. In Figure 4, the 2009 curve lies above the 2006 curve, showing a considerable improvement in the equity state of CT diffusion in Shaanxi, while there was no obvious change in Zhejiang or Hunan as shown in Figures 3 and 5.

**Figure 6:**
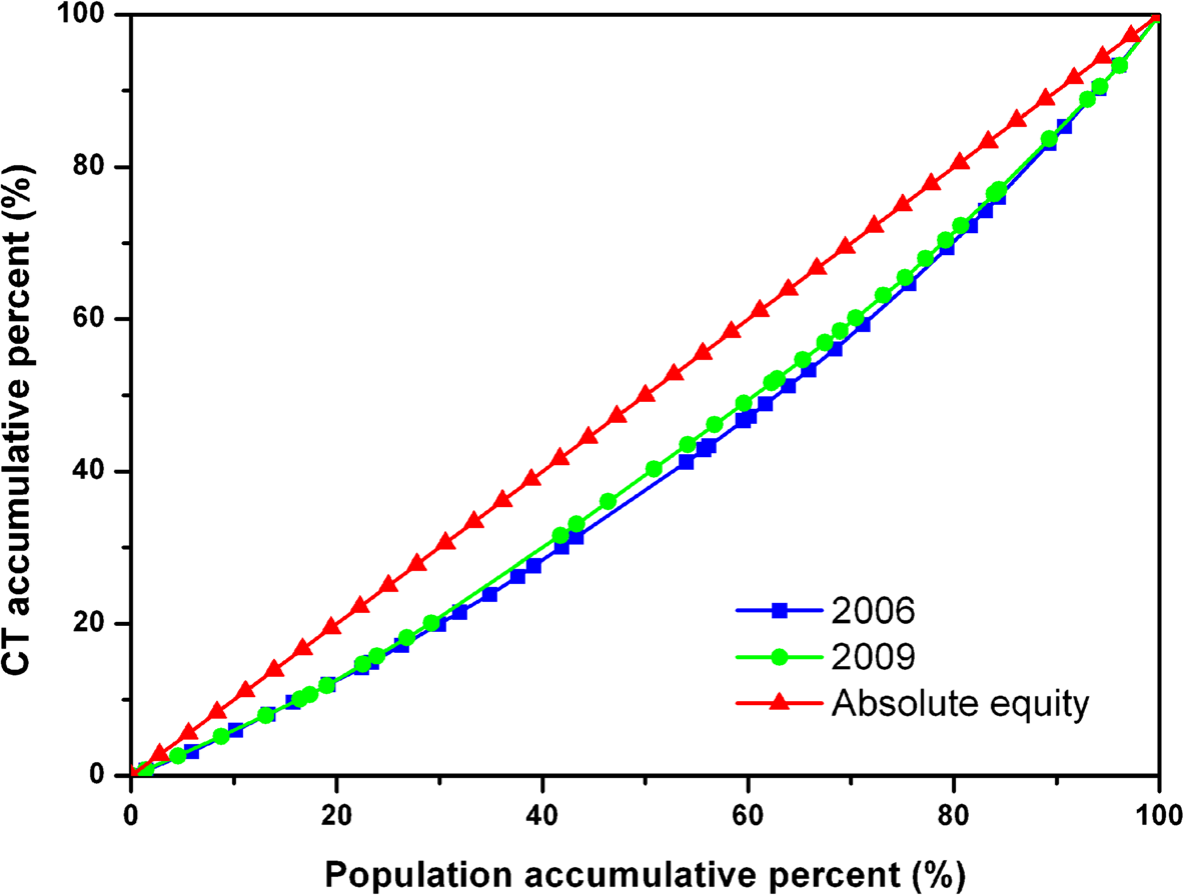
Lorenz Curve of MRIs in all cities of Zhejiang.

**Figure 7:**
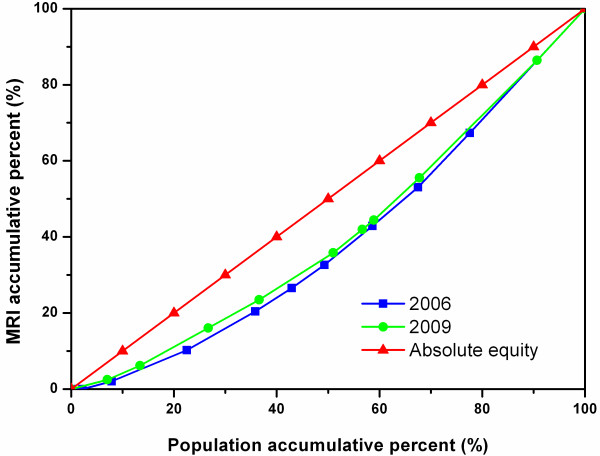
Lorenz Curve of MRIs in all cities of Shaanxi.

**Figure 8:**
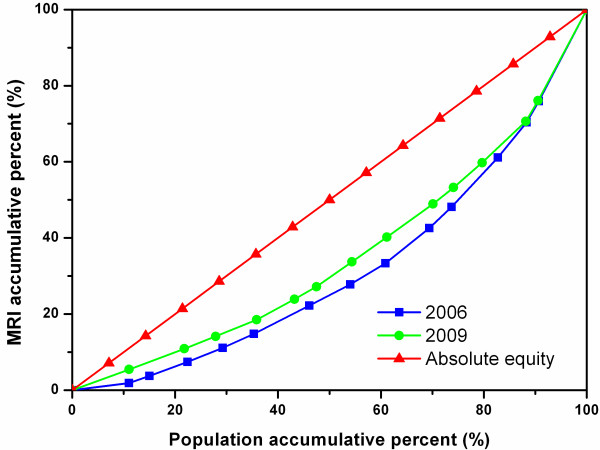
Lorenz Curve of MRIs in all cities of Hunan.

Figures 6, 7, and 8 show the diffusion of MRIs in Zhejiang, Shaanxi, and Hunan in 2006 and 2009. All three curves of MRI in 2009 lie above the ones in 2006, showing the equity state of MRIs has improved in these sites. Among the three figures, it is worth noting that the two curves in Figure 8 separate from each other most obviously, indicating that Hunan has had the biggest improvement in equity from 2006 to 2009 among the study sites. However, the curves of Figure 8 are bent furthest away from the diagonal line, which means that, despite the biggest improvement, the equity status of MRIs in Hunan was the worst in the three regions.

### Gini coefficients of CTs and MRIs in the study sites

Table 3 shows that the Gini coefficients of CTs were smaller than 0.2 either for the entire sample or for each study site in 2006, indicating low inequality based on criteria set by prior research [41,42]. The Gini coefficients of CTs dropped slightly from 2006 to 2009 for the entire sample and for each of the study sites, indicating slight improvement in the equity status of CT diffusion. Shaanxi had the biggest decline in the Gini coefficient, which reached 0.09 in 2009, the lowest among the study sites.

**Table 3:** Gini coefficients of CTs and MRIs in study sites

**Gini coefficient**	**CT**		**MRI**	
	**2006 (equity status)**	**2009 (equity status)**	**2006 (equity status)**	**2009 (equity status)**
All cities in Zhejiang	0.14 (low inequality)	0.13 (low inequality)	0.26 (moderate inequality)	0.21 (moderate inequality)
All cities in Shaanxi	0.15 (low inequality)	0.09 (low inequality)	0.22 (moderate inequality)	0.19 (low inequality)
All cities in Hunan	0.16 (low inequality)	0.15 (low inequality)	0.36 (high inequality)	0.29 (moderate inequality)
Entire sample (not including Shanghai)	0.18 (low inequality)	0.16 (low inequality)	0.35 (high inequality)	0.29 (moderate inequality)
Entire sample (including Shanghai)	0.17 (low inequality)	0.15 (low inequality)	0.32 (high inequality)	0.27 (moderate inequality)

Note: the equity status was assessed using the criteria from Luebker, et al. 2010.

A similar pattern appeared for the Gini coefficients of MRIs. From 2006 to 2009, the Gini coefficients declined for the entire sample and for each of the study sites. For the entire sample, the Gini coefficient dropped from 0.32 in 2006 to 0.27 in 2009, indicating an improvement of the equity status from high inequality to moderate inequality level [41,42]. Among the study sites, Hunan had the highest Gini coefficient of MRIs in either 2006 or 2009. It also had the largest reduction in the Gini coefficient from 2006 to 2009. As a result, the differences in the Gini coefficients across the three study sites were reduced from 2006 to 2009. Table 3 also shows that the Gini coefficient of CTs was much smaller than that of MRIs for each site in each year, indicating that the equity status of CT was better than that of MRI.

### Correlation between the economic development level and the CT and MRI distribution

**Figure 9:**
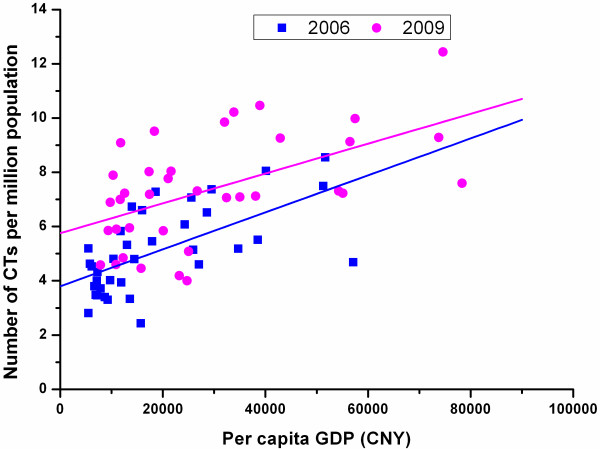
Correlation between per capita GDP and number of CTs per million population of all sample cities in 2006 and 2009.

Figure 9 shows that the number of CTs per million population was significantly positively correlated with GDP per capita across all cities in the study sites both in 2006 (r = 0.626, P < 0.00) and in 2009 (r = 0.551, P < 0.00). It also shows that the fitted 2009 line lies above the 2006 line, indicating a higher CT number for the same economic level in 2009 than in 2006. The slope of the fitted 2009 line was smaller than that of 2006, indicating that the level of CT distribution got less dependent on the economic condition across the cities in 2009.

**Figure 10:**
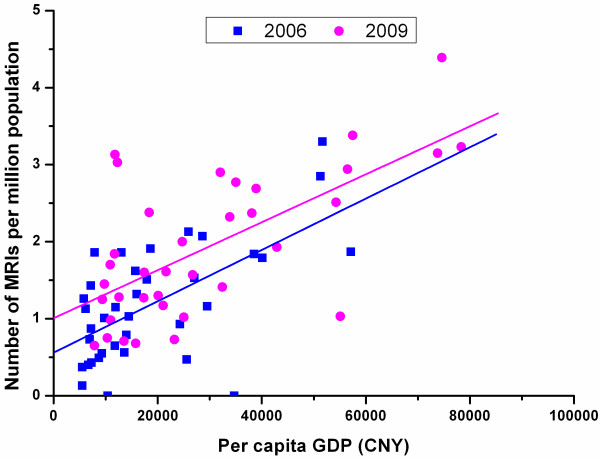
Correlation between per capita GDP and number of MRIs per million population of all sample cities in 2006 and 2009.

Figure 10 shows the significant positive correlation between the number of MRIs and GDP per capita across all cities in the study sites both in 2006 (r = 0.622, P < 0.00) and in 2009 (r = 0.664, P < 0.00). The fitted line of 2009 lies above the line of 2006, suggesting an increase in MRIs for the same economic level over the two years. The slope of the fitted 2009 line is slightly smaller than that of 2006, indicating a small improvement in the equity status of MRI distribution.

### Equity assessment by characteristics and financing of CTs and MRIs in the hospital sample

Table 4 shows the distributions of CTs by types of machine in 2009. Among the 71 sampled hospitals, there were a total of 107 CTs in 2009. The types of CTs varied substantially across the study sites with most of the CTs (76%) in Shanghai having 16-row or higher, compared with 17% in Shaanxi. The difference in the proportion of CTs with 16-row or higher was statistically significant across the study sites (*χ*^2^ test, P < 0.01).

**Table 4:** CT and MRI distributions by types of machine among the 71 sampled hospitals in 2009

**Location of hospitals**	**CTs**			**MRIs**		
	**Number of CTs investigated**	**% of CTs with 16 rows or higher**^**※1**^	**% of CTs imported**^**※2**^	**Number of MRIs investigated**	**% of MRIs with 1 · 5 T or higher**^**※3**^	**% of MRIs imported**^**※4**^
Shanghai	29	76	100	15	100	100
Zhejiang	32	53	81	18	56	89
Shaanxi	23	17	71	9	22	56
Hunan	23	39	83	12	58	100
Total	107	49	84	54	63	89

^※^*χ*2 test, P < 0.05. (^※^1: P = 0.000, ^※^2: P = 0.011, ^※^3: P = 0.000, ^※^4: P = 0.005).

Among 107 CTs we studied, 84% were imported into China. The difference in the proportion of imported CTs was statistically significant (*χ*^2^ test, P < 0.05), with all the CTs in the sample hospitals in Shanghai being imported, compared with 71% of imported CTs in Shaanxi.

Table 4 also shows the distributions of MRIs by types of machine in 2009. Among the 71 sampled hospitals, there were a total of 54 MRIs in 2009. As to the two categories of MRI machine, all the MRIs in the sampled hospitals in Shanghai had 1.5 T or higher, compared with 22% in Shaanxi. The difference in the proportion of MRIs with 1.5 T or higher was statistically significant across the study sites (*χ*^2^ test, P < 0.01).

Most of the MRIs we studied were imported (89%). Both Shanghai and Hunan had 100% imported MRIs in the sample hospitals, compared with 56% in Shaanxi. The difference across the study sites was statistically significant (*χ*^2^ test, P < 0.01).

Table 5 shows information about financing of CTs in the 71 sampled hospitals in 2009. The sample from Shanghai had the highest proportion of MRIs with government subsidies (38%), compared with no subsidies for CTs in Hunan. The difference across the study sites was statistically significant (*χ*^2^ test, P < 0.01). In terms of average cost of per CT machine, there was no statistical significance across the study sites.

**Table 5:** Financing of CTs and MRIs in the 71 sampled hospitals in 2009

**Location of hospitals**	**CTs**				**MRI**			
	**Number of CTs investigated**	**% of CTs with government subsidy**^※^	**Government subsidy as % of total CT costs**	Average cost per CT (1,000USD) and 95% CI^*^	**Number of MRIs investigated**	**% of MRIs with government subsidies**	**Government subsidy as % of total MRI costs**	Average cost per MRI (1,000USD) and 95% CI^*^
Shanghai	29	38	32	918	15	27	20	1,925
				(255; 1,081)				(1,667; 2,184)
Zhejiang	32	16	13	901	18	22	10	1,661
				(749; 1,053)				(1,293; 2,029)
Shaanxi	23	9	2	688	9	11	1	1,097
				(550; 827)				(770; 1,424)
Hunan	23	0	0	936	12	0	0	1524
				(674; 1,198)				(896; 2,153)
Total	107	17	1	867	54	17	1	1,610
				(780; 955)				(1410; 1810)

Note: Exchange rate: 1USD = 6.3CNY.

^※^*χ*^2^ test, P = 0.002 < 0.05.

^*^95% CI: 95% confidence interval.

Table 5 also shows the variation in financing of MRIs in the sampled hospitals in 2009. Although the sampled hospitals in Hunan did not receive government subsidies for MRIs, the difference in the proportion of MRIs with government subsidies was not statistically significant across the study sites (*χ*^2^ test, P > 0.05). In terms of MRI machine costs, the average costs per MRI in the sampled hospitals in Shanghai was US$1,925, 000, the highest among the study sites, which was significantly higher than that in Shaanxi (US$1,097, 000). The statistical significance was based on the 95% confidence intervals of the average costs.

Table 6 compares financing of CTs and MRIs between secondary and tertiary hospitals in 2009. The average cost per MRI in the sampled secondary hospitals was US$1,230,000, which was significantly lower than that in tertiary hospitals (US$1,852,000). The statistical significance was based on the 95% confidence intervals of the average costs. There was no significant difference between the two levels of hospitals in terms of other indicators, such as CTs with government subsidies, average CT costs, and MRIs with government subsidies.

**Table 6:** Financing of CTs and MRIs in the sample secondary and tertiary hospitals in 2009

**Location ofhospitals**	**CTs**				**MRI**			
	**Number of CTs investigated**	**% of CTs with government subsidy**	**Government subsidy as % of total CT costs**	Average cost per CT (1,000USD) and 95% CI^*^	**Number of MRIs investigated**	**% of MRIs with government subsidies**	**Government subsidy as % of total MRI costs**	Average cost per MRI (1,000USD) and 95% CI^*^
Secondary hospitals	45	11	7	779	18	28	29	1230
				(689; 869)				(900; 1,543)
Tertiary hospitals	62	21	18	932	36	11	4	1,852
				(796; 1,067)				(1,627; 2,077)
Total	107	17	14	867	54	17	10	1,610
				(780; 955)				(1410; 1810)

Note: Exchange rate: 1USD = 6.3CNY.

^*^95% CI: 95% confidence interval.

## Discussion

We found that China had lower numbers of CTs and MRIs per million population in 2009 than most of the selected OECD countries while the increases in its CT and MRI numbers from 2006 to 2009 were higher than most of the OECD countries. These findings suggest that China, which is the largest developing country, still lags behind the developed countries in high-technology medical equipment, such as CTs and MRIs. On the other hand, with its rapid economic development and large population, the numbers of CTs and MRIs in China are expected to increase substantially in the coming years.

We also found that the equity status of CT distribution remained at low inequality level in both 2006 and 2009. In comparison, the equity status of MRI distribution improved from high inequality in 2006 to moderate inequality in 2009. Overall, the equity status of CTs was better than that of MRIs in either 2006 or 2009. This may be related to the facts that CTs are relatively less expensive than MRIs and that they are widely used in both tertiary and secondary hospitals [46]. In comparison, MRIs are mainly purchased by tertiary hospitals and only rarely by secondary hospitals.

The degree of equity improvement also varied among different study sites. Among the study sites, Shaanxi had the largest decrease in the Gini coefficient of CTs, reaching 0.09 in 2009, the best equity status in the study sample. It is worth noting that the Gini coefficient of MRI in Hunan dropped from 0.36 to 0.29 from 2006 to 2009, indicating that the equity status was enhanced from high inequality to moderate inequality. It is worth noting the equity improvement in Shaanxi and Hunan, both of which are large rural provinces with medium income level in China.

Despite the improvement in equity status of CTs and MRIs in some of the study sites, we found that the distributions of CTs and MRIs were significantly positively correlated with economic development level across all cities in the four study sites in either 2006 or 2009. Our analysis also revealed that Shanghai, the study site with the highest level of economic development, had more advanced CT and MRI machines, imported more CTs and MRIs, and received higher government subsidies for these two types of equipment. Together, these findings indicate that the CT and MRI distributions, especially the distribution of more advanced machine models, were significantly influenced by the economic development levels and government subsidies among Chinese regions.

The Gini coefficients found in our study are generally consistent with the literature. We found that the Gini coefficients for CT varied from 0.09 to 0.18, similar to the range of 0.06 to 0.26, which were reported by previous studies [8,9,13]. In terms of the Gini coefficients for MRI, we found a range of 0.19 to 0.36, similar to the range of 0.12 to 0.32 known in the literature [8,9,16]. Our findings of relatively low numbers of CT and MRI in the UK and high numbers of CT and MRI in Japan are also consistent with prior international studies [23,47]. Our panel analysis revealed the changes in CT and MRI equity status across regions over time, offering an improvement over the current literature, which typically used cross-sectional data to examine the equity status [9,26].

Our study has a number of strengths, such as using panel data to provide updated information about CTs and MRIs in China, conducting analyses at international, provincial, city, and hospital levels, and examining detailed information about these two types of equipment. However, our study sites, which were drawn from different regions, do not include some of the least developed provinces in China, such as Qinghai and Gansu. It remains an empirical question for future studies to examine whether including those least developed provinces in the sample will result in substantial changes in the equity status of CT and MRI distribution. This study used data about the capital city and one prefecture-level city in every study province, and the results could not be extrapolated to all prefecture cities within the provinces, nor could be extrapolated to counties, since there are several prefectures in one province and numerous counties in each prefecture. This study does not examine the causal effects of the Chinese CON policies on the distributions of CTs and MRIs. To identify the causal effects, future studies need to use a pre-post study design, such as a comparison between 2000–2005 and 2006–2010, to evaluate whether the increases of CTs and MRIs after the CON policies significantly differ from the trends in CT and MRI growth before the policies. This study analyzed the 2006 and 2009 data, and the results did not reflect changes in CT and MRI distribution in the most recent years. Our results can be considered as baseline information for future studies to evaluate whether the distribution changes significantly after China’s central government initiated the nationwide health care reform in 2009. This study also does not examine equity in the utilization of CTs and MRIs. International studies have confirmed that utilization-based analyses of health care resource, such as the utilization status of CT and MRI, provide a more accurate assessment than simple population-based analyses, and therefore have the potential to help improve the resource allocation efficiency [47,48]. Future studies in China need to be conducted to examine whether those who need CT or MRI examinations actually have their needs met and to see whether those who do not need CT or MRI scans are not involved in the overuse of these two types of equipment. While this study has examined equity in the distribution of CT and MRI, both of which are inputs of health care, it remains an important topic for future studies to examine how the inequality in CT and MRI distribution leads to inequality in health care outputs, such as health care quality and population health.

## Conclusions

To our knowledge, this paper is the first study that examines the CT and MRI distributions not only by the numbers of machines but also by the machines’ characteristics. Our study findings have important policy implications. First, the findings of rapid increases in the numbers of CTs and MRIs have huge financial implications for hospital budgets in China. Policymakers and researchers need to monitor the trends of how Chinese hospitals are paying for these types of high-technology equipment and how they recoup the costs. Given the concern that Chinese hospitals earn a large percentage of their revenues from using high-technology medical equipment, such as CTs and MRIs [2,28], the rapid increases in the numbers of CTs and MRIs found among the study sites may be a sign of a medical arms race among Chinese hospitals and may imply overuse and/or inequitable use of these types of medical equipment in China.

Second, we found that the substantial government subsidies in wealthy regions such as Shanghai helped hospitals purchase more advanced models of CTs and MRIs. This finding suggests that, to help improve equity, the central government may want to invest directly in the high-technology medical imaging equipment in poor provinces, such as those provinces in the middle and west regions, as part of its ongoing financial support to the poor provinces.

Third, our findings of the improved equity status in CT and MRI distribution over the study period, especially among the large rural provinces such as Shaanxi and Hunan, are encouraging. The improved equity status may be due to the policy initiative, Chinese CON, implemented since 2005. To the extent that future studies identify which specific policies implemented since 2005 contribute to the improved equity in the study sites, policymakers may want to consider scaling up the policy implementation in a new round health reform.

Fourth, our findings of inequality in CT and MRI distributions by types of machine suggest that policymakers may want to revise the current medical equipment policy in China, which focuses on total volume contrl through a quota system of the CON programs. While the policy may help slow the increase in the numbers of CTs and MRIs and improve their equitable distributions, it does not address characteristics of machine. Thus, the policy needs to be revised to consider not only the numbers of CTs and MRIs but also the machine characteristics, especially the advanced models.

Finally, we found that the vast majority of CTs and MRIs were imported and only small percentages of CTs and MRIs were made by domestic Chinese firms, indicating that China is still at a low level of high-technology medical equipment development. Since most of the CTs and MRIs are imported, Chinese hospitals have to pay higher prices for the imported machines (compared to the domestically-made machines) and to make substantial efforts to train staff to operate and maintain the machines. Taken together, the large numbers of imported CTs and MRIs pose a challenge not only for Chinese hospitals but also for Chinese policymakers, who regulate the manufacture, import and use of high-technology equipment. Given the Chinese government’s emphasis on technology innovation [49], including medical technology, policymakers and researchers need to monitor trends in the production and distribution of CTs and MRIs and examine the implications for hospital financing.

## Endnotes

^a^Row is the unit of the slices of detectors. The higher the rows, the faster the scan detectors and the clearer the picture a CT can produce

^b^T is the symbol of tesla, which is the SI derived unit of magnetic flux density. One tesla is equal to one weber per square meter. It is the unit of strength of medical magnetic resonance imaging systems in practice

## Abbreviations

CT: Computed tomography; MRI: Magnetic resonance imaging; MOH: The Ministry of health; CON: Chinese certificate of needs; GDP: Gross domestic product; OECD: Organization for economic co-operation and development

## Competing interests

The authors declare that they have no competing interests.

## Authors’ contributions

All authors participated in the conception and design of the report and took responsibility for the integrity of the work as a whole. YYC conceived and designed the study, coordinated data collection, directed data analysis, helped draft the manuscript and revised the manuscript. HY provided insightful suggestions to study design, directed data analysis and revised the manuscript critically for important intellectual content. DH took responsibility for acquisition of data, had full access to all the data in the study, took responsibility for the integrity of the data, conducted data analysis, and drafted the manuscript. All authors approved the final version to be published.
